# Deuteration‐Induced Energy Level Structure Reconstruction of Carbon Dots for Enhancing Photoluminescence

**DOI:** 10.1002/advs.202308523

**Published:** 2024-05-30

**Authors:** Zimin Yao, Xiaokun Wen, Xia Hong, Ran Tao, Feifei Yin, Shuo Cao, Jiayi Yan, Kexin Wang, Jiwei Wang

**Affiliations:** ^1^ College of Physics Liaoning University Shenyang 110036 China; ^2^ Key Laboratory of UV‐Emitting Materials and Technology (Northeast Normal University) Ministry of Education Changchun 130024 China

**Keywords:** carbon dots, deuteration, energy level structure, photoluminescence, white‐light‐emitting diodes

## Abstract

Constrained by a limited understanding of the structure and luminescence mechanisms of carbon dots (CDs), achieving precise enhancement of their photoluminescence (PL) performance without altering the emission wavelength and color remains a challenge. In this work, a deuterated CD is first achieved by simply replacing the reaction solvent from H_2_O to D_2_O. The substitution of D atoms for H atoms is not limited on the surface but also within the internal structure of CDs. Deuteration affects the formation of the *π*‐conjugated network structure by altering the content of sp^2^ carbon and sp^3^ carbon, ultimately inducing a reconstruction for energy level structure of CDs. Both the intrinsic state and surface state emission, including quantum yield, emission intensity and lifetime, are significantly enhanced after deuteration. It benefits from the reduction in non‐radiative transitions, since the lowered vibrational frequencies of D atoms and optimized local energy level distribution in CDs structure. The deuterated CDs are applied in the fabrication of white‐light‐emitting diodes to show their application potential. This work provides a highly versatile route for improving and controlling photoluminescence performance of CDs and has opportunities to guide the development of CDs for practical applications.

## Graphical abstract

Deuterated carbon dots (CDs) are firstly achieved by simply replacing the reaction solvent from H_2_O to D_2_O. The substitution of D for H induces a reconstruction of the CD energy level structure. Both the intrinsic state and surface state emission, including quantum yield, emission intensity and lifetime, are significantly enhanced after deuteration. This work provides a highly versatile route for improving and controlling photoluminescence performance of CDs.

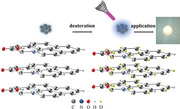

## 1 Introduction

Carbon dots (CDs) have emerged a promising class of carbon‐based luminescence material in recent years, garnering attention due to their distinctive advantages, such as fascinating chemical inertness, high optical and thermal stability, great water dispersibility and biocompatibility.^[^ ^1^, ^2^, ^3^, ^4^, ^5^ ^]^ These advantages endow CDs with potential applications in optoelectronic devices, biosensing, photocatalysis and energy storage.^[^ ^6^, ^7^, ^8^, ^9^ ^]^ Despite the tremendous potential of CDs, the complexity of structure and composition still limits a comprehensive understanding for the structure‐property relationship of CDs. The exact photoluminescence (PL) mechanism of CDs remains unsettled. It hinders the broader practical applications of CDs. The enhancement and modulation of luminescence performance thus lack highly universal approaches.

Currently, the mainstream understanding of CDs luminescence mechanism primarily involves the synergetic contribution of intrinsic state luminescence and surface state luminescence.^[^ ^10^, ^11^, ^12^, ^13^ ^]^ The intrinsic state luminescence is believed to originate from the conjugated *π*‐bond networks within the carbon core and the surface state luminescence is attributed to surface defects and functional groups.^[^ ^14^, ^15^, ^16^ ^]^ Plenty of works have confirmed the ability to manipulate PL properties through modulating surface functional groups and doping heteroatoms.^[^ ^17^, ^18^ ^]^ However, in the pursuit of improving PL performance of CDs, surface modification and doping often accompany the changes in emission wavelength and color. It is challenging to achieve enhancement in PL performance of CDs while maintaining consistency on optical properties. The development of versatile routes for precise optimization of PL performance holds significant practical relevance.

Deuteration is considered one of the effective approaches to enhance the performance of carbon‐based luminescence materials.^[^ ^19^, ^20^, ^21^ ^]^ The emission quenching effect can be inhibited by deuteration in some organic materials and consequently improved the overall performance.^[^ ^22^, ^23^, ^24^, ^25^ ^]^ The substitution of H atoms with D atoms lead to alterations in the vibrational properties of molecules and intermolecular interactions. For CDs, it signifies a comprehensive alteration in orbital hybridization and electronic band structure. The induced effects from D substitution on covalent bond lengths and angles can lead to the reconstruction of the spatial geometry of the *π*‐conjugated network. The changes in vibrational frequencies alter the distribution of local electronic states in CDs. These factors have the opportunity to ameliorate the local energy level distribution in CDs structure, thereby enhancing their PL performance without the introduction of other dopant atoms and additional functional groups.

Herein, the deuteration of CDs were achieved by simply replacing H_2_O with D_2_O as the solvent in the hydrothermal reaction. The substitution of D for H is not limited to the surface but also extends to the internal *π*‐conjugated network of CDs. The systematic analysis of IR, and XPS indicates that deuteration induced the restructure of CDs by adjusting the ratio of sp^2^ carbon to sp^3^ carbon in their structure and electronic bands. The deuteration‐induced enhancements in the surface state and the intrinsic state luminescence of CDs were observed through measurements of emission intensity, quantum yield, and luminescence lifetime. The white light‐emitting diodes (WLED) have been fabricated by employing the deuterated CDs to demonstrate their potential applications. These results thus present a highly versatile pathway for regulating PL performance on CDs, which may act as a guide for diverse promising applications based on CDs.

## 2 Results and Discussion

**Scheme 1:**
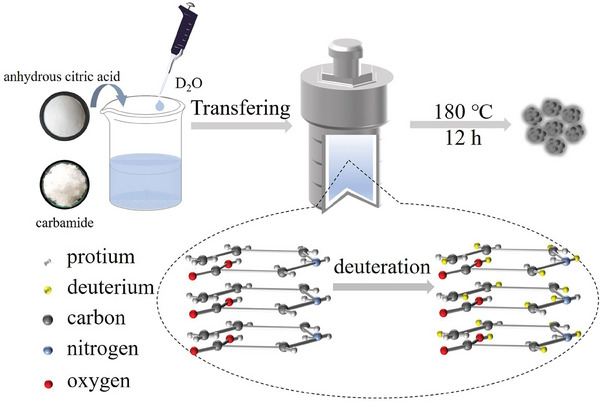
Mechanism of the substitution of H atoms with D atoms.

The CDs were prepared via the classical hydrothermal method (**Scheme** **1**). The deuteration of CDs was achieved by partially or completely replacing the reaction solvent with D_2_O. The samples are named as D_0_‐CDs, D_0.2_‐CDs, D_0.3_‐CDs, D_0.5_‐CDs, D_1_‐CDs, according to the specific proportion of D_2_O in the reaction solvent.

**Figure 1:**
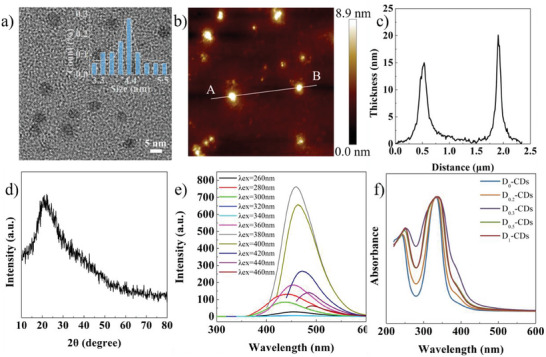
a) HRTEM image and size distribution of CDs. b) AFM image of CDs. c) Thickness distribution of CDs. d) XRD pattern of CDs. e) PL spectra of CDs under different excitation wavelengths. f) UV–vis absorption spectra of CDs.

The TEM image of CDs is shown in **Figure** **1**a. The CDs have an average size of 4.28 ± 0.21 nm. The morphology and thickness distribution of CDs are further characterized using AFM (Figure 1b,c). The size and thickness of the CDs are measured as ≈15.02–20.12 nm. Figure 1d is the XRD pattern of CDs. The band centered ≈23.9° is assigned to the amorphous carbon structure of CDs.^[^ ^26^ ^]^ The prepared CDs exhibit the excitation‐dependent PL behavior. The emission spectra of CDs acquired from various excitation wavelengths are presented in Figure 1e (the concentration of CDs dispersion is 1 mg mL^−1^). The center of emission band undergoes a redshift from 449 to 491 nm as the excitation wavelength changes from 360 to 460 nm. It arises from the synergistic effect of surface state and intrinsic state emission mechanisms.^[^ ^27^, ^28^ ^]^ The UV–vis absorption spectra of five CDs samples are shown in Figure 1f. The absorption bands at 240 and 340 nm are assigned to *π*→*π* ^*^ transition and *n*→*π* ^*^ transition.^[^ ^29^, ^30^ ^]^ The absorption shoulder from 350 to 400 nm is ascribed to the surface non‐conjugated functional groups and defects. D_0.3_‐CDs have the highest absorbance of the shoulder among the samples. The contents of D elements in deuterated CDs are tested by isotope mass spectrometry and summarized in **Table** **1**. The max isotope abundance of samples reaches 3.15%.

**Table 1:** The content of D elements in CDs.

Sample	D atom [%]
H_2_O	0.0147 ± 0.00137
D_0_‐CDs	0.0148 ± 0.00115
D_0.2_‐CDs	1.36 ± 0.0529
D_0.3_‐CDs	3.04 ± 0.173
D_0.5_‐CDs	2.84 ± 0.0717
D_1_‐CDs	3.15 ± 0.216

**Figure 2:**
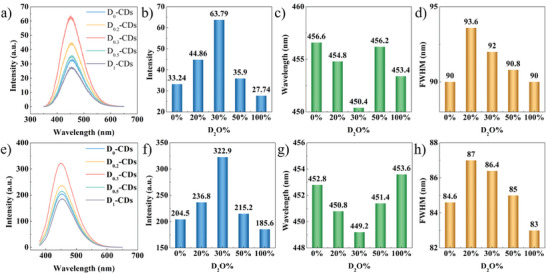
a,e) PL spectra of CDs and deuterated CDs dispersion under 260 and 360 nm excitation (concentration of CDs in H_2_O is 1 mg mL^−1^). b,f) Emission intensity at 260 and 360 nm of CDs and deuterated CDs. c,g) Emission wavelength of CDs and deuterated CDs under 260 and 360 nm excitation. d,h) FWHM of CDs and deuterated CDs under 260 and 360 nm excitation.

Deuteration modulates the PL performance of CDs by affecting two emission mechanisms. **Figure** **2**a presents the emission spectra of CDs under 260 nm excitation. It is a typical emission dominated by intrinsic states. The emission intensity of CDs acquired a maximum enhancement of up to 92% after deuteriation (Figure 2b). The absolute quantum yield is improved from 9.9% to a maximum of 12.2%. The enhancement of emission intensity is accompanied by a slight blue shift of the emission band center and a broadening of the full width at half maximum (FWHM). It reflects that deuteriation introduces extra luminescence energy levels, ameliorating the energy level distribution in CDs. Emission intensity increases up to 30% D incorporation in CD. On further increase in D, the intensity is observed to decrease. The FWHM of emission bands exhibits a continuous narrowing trend with the rise of the D_2_O content. They might stem from the contribution of deuteration on the surface state emission of CDs. The emission spectra are recorded under excitation at 360 nm to explore the surface state emission of CDs (Figure 2e). The emission intensity of CDs reaches the maximum when the D_2_O proportion in the reaction system is 30% (Figure 2f). It exhibits a 58% enhancement after deuteriation. The absolute quantum yield is increased from 10.9% up to a maximum of 25.3%. The enhancement in emission intensity is accompanied by a slight blue shift in the emission maxima and a widening of FWHM. It originates from the modulation of the surface state energy levels by deuteriation. As the D_2_O content in the reaction system increases, the prepared CDs exhibit narrower FWHM. The emission band center of prepared CDs shows no significant shift. The chemical composition and structure of CDs are studied through infrared spectroscopy.

**Figure 3:**
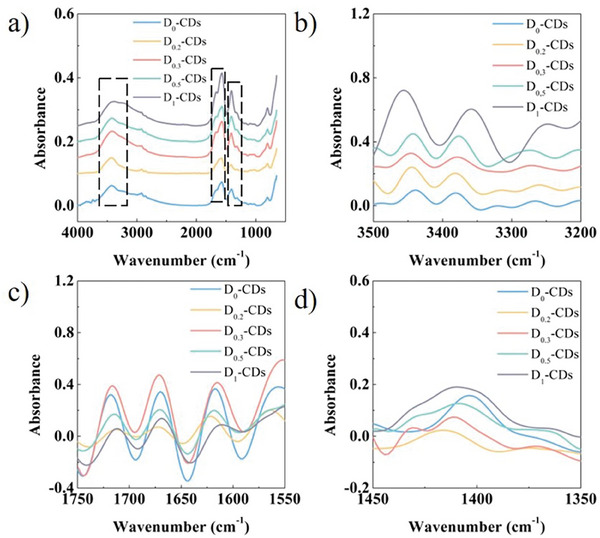
a) FT‐IR spectra of CDs and deuterated CDs. b–d) FT‐IR deconvolution spectra of CDs and deuterated CDs.

The Fourier transform infrared (FTIR) spectra of CDs are shown in **Figure** **3**a. The broad absorption bands can be observed at 3448 cm^−1^. It is attributed to the stretching vibration of hydroxyl groups and amino groups. The peaks at 1716, 1670, and 1620 cm^−1^ are attributed to the stretching vibration of carbonyl groups, carbon–nitrogen double bonds and carbon–carbon double bonds, respectively. The peak at 1415 cm^−1^ is attributed to stretching vibration band of carbon–nitrogen bonds. The central wavenumber and bandwidth of these characteristic absorption of CDs show some changes after deuteration. Fourier deconvolution was performed on the FTIR spectra to obtain more spectral details. The Fourier deconvolution spectrum of stretching vibration of hydroxyl groups and amino groups is shown in Figure 3b. Their bandwidth and central wavenumbers manifest clear change in CDs. Figure 3c is the Fourier deconvolution spectra in 1750–1550 cm^−1^. The bands are assigned to the stretching vibration bands of carbonyl groups, carbon–nitrogen double bonds and carbon–carbon double bonds. The central wavenumbers of the groups show a blue shift. The Fourier deconvolution spectrum in 1450–1350 cm^−1^ is shown in Figure 3d. The vibrational frequency range of carbon–nitrogen bonds is broadened. The center wavenumber of carbon–nitrogen bonds decreases. These changes in bandwidth and band position are caused by the substitution of H atoms with D atoms on the bonds. The heavier D atoms lead to a decrease in the vibrational frequency of the relevant chemical bonds and functional groups and introduce additional molecular vibrational modes. Since the infrared spectra were tested in transmission mode, these results indicate that substitution of H atoms with D atoms occurs not only on the surface but also within the internal structure of CDs.

**Figure 4:**
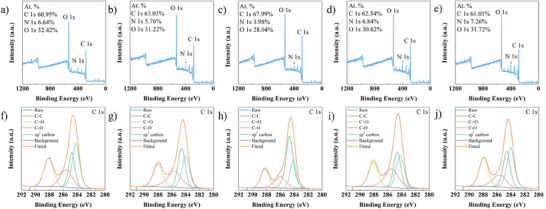
The XPS survey of CDs. D_0_‐CDs a), D_0.2_‐CDs b), D_0.3_‐CDs c), D_0.5_‐CDs d) and D_1_‐CDs e). The high‐resolution C1s XPS spectra of D_0_‐CDs f), D_0.2_‐CDs g), D_0.3_‐CDs h), D_0.5_‐CDs i), and D_1_‐CDs j), respectively.

The CDs were characterized by XPS spectra to further explore the impact of deuteration on PL performance from the perspective of electronic band structure. CD samples all have three typical peaks at 531.6, 399.8, and 284.6 eV (**Figure** **4**a–e). They are assigned to O 1s, N 1s, and C 1s, respectively. The N1s high‐resolution XPS spectra are shown in Figure 4. The pyrrolic N (399.5 eV) is found in five CDs. The binding energy of pyrrolic N is almost unchanged. The high‐resolution C 1s spectra of CDs are shown in Figure 4f–j. It is fitted into four peaks at 284.0, 284.6, 287.9, and 285.5 eV attributed to sp^2^ carbon, C─C, C═O, and C─O/C─N, respectively. The area variation of sp^2^ carbon band is shown in Figure S4b (Supporting Information). D_0.3_‐CDs has the lowest area of sp^2^ carbon. The changes of binding energy about the C species showed in Figure S4a (Supporting Information). The binding energy of C═O and C─O/C─N in deuterated CDs is higher than that in CDs. D atoms with higher mass and atomic radius leads to changes on the vibrational frequency and length of chemical bonds in CDs. The construction of conjugated structures based on σ and *π* bonds is also affected. It implies that the deuterium substitution occurring in the formation of CDs induces a restructuring of the energy band structure and the localization effect between atoms.

**Figure 5:**
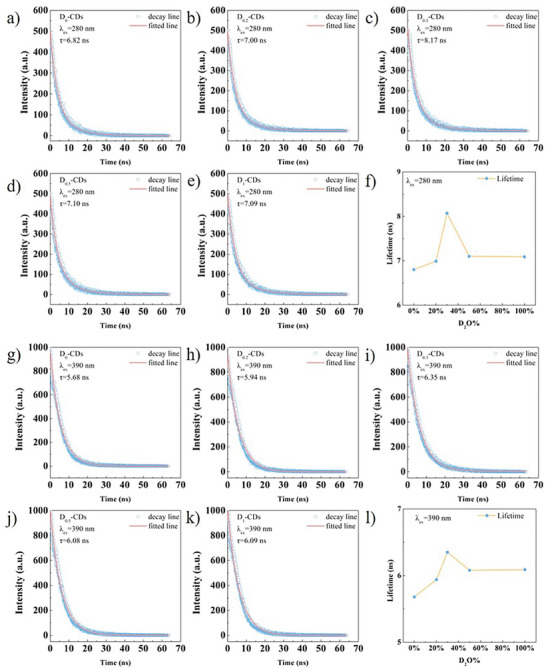
The time‐resolved decay spectra of CDs under excitation at a–e) 280 nm and g–k) 390 nm. The variation of lifetime about five CDs under excitation at f) 280 nm and l) 390 nm.

Time‐resolved PL decay curves were measured to study the excited‐state radiative relaxation process of the CDs. The average lifetimes (*τ*) under excitation at 280 and 390 nm are shown in **Figure** **5** and **Table** **2**. They are obtained by biexponential fitting. The decay process consists of the fast component *τ*_1_ and the slow component *τ*_2_. *τ*_1_ usually represents the non‐radiative composite and surface state emission.^[^ ^31^, ^32^, ^33^ ^]^ *τ*_2_ is attribute to the radiative recombination of the intrinsic state.^[^ ^34^, ^35^, ^36^ ^]^ The ratios of *τ*_1_ and *τ*_2_ exhibit discrepancy under 280 and 390 nm excitations. It indicates that the presence of two emission mechanisms (intrinsic state emission and surface state emission). The lifetime of CDs improves from 6.82 ns to a maximum of 8.17 ns (280 nm excitation) and from 5.68 to 6.35 ns (390 nm excitation) after deuteration. It illustrates that the deuteration of CDs reduces the likelihood of the non‐radiative recombination during the de‐excitation process. Both the radiative transition efficiency of the intrinsic states and the surface states are promoted. The amplitude A_1_ and A_2_ of the time‐resolved decay lifetime of CDs are recorded in Table 2. The increase of amplitude ratio A_1_/A_2_ signifies a change in the relative contributions of the intrinsic state and the surface state emissions after deuteration. The change implies that deuteration leads to adjustments in the local electronic energy level distribution in CDs. Additionally, the absolute quantum yields of CDs and deuterated CDs were measured and recorded in Table S1 (Supporting Information). The improvement of quantum yields after deuteration is also believed to stem from the decrease on vibrational frequency of functional groups in the structure of CDs as a result of substituting D atoms. It implies that the decrease in the probability of non‐radiative transitions. The additional mass introduced by D substitution may alter the electron‐phonon interactions and hydrogen bonding interactions among *π*‐conjugated carbon structures in CDs. It can optimize the local energy level distribution in CDs structure to enhance radiative transition efficiency.

**Table 2:** Survey of PL lifetimes of the CDs under 280 and 390 nm excitation.

Excitation	Category	*τ* [avg]	χ^2^	*τ*_1_ [ns]	A_1_ [%]	*τ*_2_ [ns]	A_2_ [%]	A_1_/A_2_
280 nm	D_0_‐CDs	6.82	0.9931	3.78	78.72	10.77	21.28	3.70
	D_0.2_‐CDs	7.00	0.9923	3.97	80.54	11.37	19.46	4.14
	D_0.3_‐CDs	8.17	0.9905	4.46	88.36	16.00	11.64	7.59
	D_0.5_‐CDs	7.10	0.9916	3.91	79.25	11.31	20.75	3.82
	D_1_‐CDs	7.09	0.9930	4.04	80.71	11.56	19.29	4.18
390 nm	D_0_‐CDs	5.68	0.9940	5.68	50.00	5.68	50.00	1.00
	D_0.2_‐CDs	5.94	0.9936	5.94	50.00	5.94	50.00	1.00
	D_0.3_‐CDs	6.35	0.9970	6.35	50.00	6.35	50.00	1.00
	D_0.5_‐CDs	6.08	0.9957	6.08	50.00	6.08	50.00	1.00
	D_1_‐CDs	6.09	0.9940	6.09	50.00	6.09	50.00	1.00

**Figure 6:**
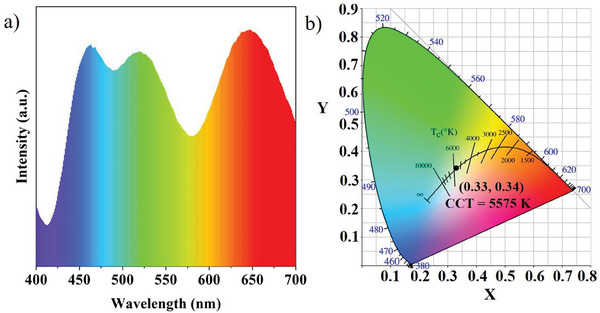
The corresponding emission spectrum a) and the CIE chromaticity coordinate b) of the WLED.

The deuterated CDs with enhanced PL performance are used in WLEDs to showcase their practical utility. The deuterated CDs were initially coated with silica to prevent aggregation quenching. The warm WLED was made by encapsulating a UV chip (380 nm) in the composites containing the D_0.3_‐CDs@SiO_2_ and commercial powders. The proportions of D_0.3_‐CDs@SiO_2_ were adjusted to tune the emission to pure white with CIE color coordinates of (0.33,0.34). The spectrum of fabricated WLED consist of three emission bands peaked at 460, 530, and 650 nm (**Figure** **6**a). The color temperature and color rendering index (CRI) were 5575 K and 81.5 (Figure 6b). It is superior to existing commercial WLEDs. The result demonstrates the potential application of the deuterated CDs in the preparation of high‐performance WLEDs.

## 3 Conclusion

In summary, CDs were prepared and deuterated using a simple hydrothermal method. Deuteration adjusted the energy level structure of CDs by altering the interactions between conjugated units. Both the intrinsic state and surface state emission, including quantum yield, emission intensity and lifetime, were significantly enhanced. It is assigned to the contribution of the reduction in non‐radiative transitions, since the lowered vibrational frequencies of D atoms and enhanced. The results provide a novel approach for improving the PL performance of CDs, which may promote their practical applications.

## 4 Experimental Section

### Chemicals

Anhydrous citric acid, deuterium, 1‐pentanol, polyvinylpyrrolidone (PVP, MW = 40000), tetraethylorthosilicate (TEOS), sodium citrate, ammonium hydroxide and ethanol were purchased from Aladdin. Carbamide were purchased from Tianjin Reference Chemical Reagent Co., LTD. Deionized water (18 MΩ cm^−1^) was used in all aqueous solutions. All of the above chemicals were used without further purification.

### Preparation of CDs and Deuterated CDs

Anhydrous citric acid (4.00 g) and carbamide (3.76 g) were dissolved in deionized water (20 mL). The mixed solution was sealed into the reaction kettle and reacted at 180 °C for 12 h. After cooling to room temperature naturally, the reaction solution was centrifuged to remove the precipitates. The supernatant was dialyzed by deionized water (1 L) for 48 h. The dialyzed solution was concentrated to 50 mL by the rotary evaporator. The concentrated solution was frozen by liquid nitrogen and lyophilized to obtain the powder products. The powder was stored in the dry and dark environment for further use.

### Fabrication of WLEDs

CDs were initially encapsulated with SiO_2_ before the WLED applications. The D_0.3_‐CDs@SiO_2_ were synthesized by an emulsion‐droplet‐based method.^[^ ^37^, ^38^ ^]^ A emulsion was first obtained by mixing PVP (2 g), sodium citrate (0.0054 g), H_2_O (0.8 mL), 1‐pentanol (20 mL), ammonium hydroxide (1 mL) and D_0.3_‐CDs (0.02 g). After vortex mixing until clarification, TEOS (0.2 mL) was added into the emulsion. The emulsion was left to react at 50 °C for 4 h after thorough re‐mixing. The products were centrifugated and washed by deionized water. The white precipitate was dried to collect for the next application.

UV‐LED chips (emission wavelength centered at 380 nm) were used as the substrates for the fabrication of WLED. The powders of D_0.3_‐CDs@SiO_2_ were mixed with green/red commercial luminescence powders (Ce‐YAG). Then they were encapsulated with epoxy resin adhesive on the UV chips. The chips were dried at 150 °C for 1 h.

### Characterization

The X‐ray diffraction (XRD) pattern of CDs were acquired using an automatic X‐ray diffractometer system (TD‐3500, Tongda). The morphology of CDs was characterized by the transmission electron microscope and atomic force microscopy (TEM, 2100F, JEM and AFM, Icon, Bruker). The UV–vis spectrum of CDs was obtained by a spectrophotometer (UH‐4150, Hitachi). The fluorescence emission spectra of CDs were recorded using fluorescence spectrometer (F‐7000, Hitachi). Time‐resolved fluorescence spectra of CDs were recorded by a transient optical testing system (Quanta Master 400, Photon Technology International). Fourier transform infrared (FTIR) transmittance spectra of CDs were recorded with Nicolet IS10 spectrometer under the liquid nitrogen environment. X‐ray photoelectron spectra (XPS) of CDs were characterized by ThermoFisher 250xi X‐ray spectrometer. Atomic force microscope (AFM) was used to measure the morphology of CDs in air with a Bruker Dimension Icon instrument.

## Conflict of Interest

The authors declare no conflict of interest.

## Supporting information

Supporting Information

## Data Availability

Research data are not shared.
