# Supplementary material for: Deuteration‐Induced Energy Level Structure Reconstruction of Carbon Dots for Enhancing Photoluminescence
Source: Adv Sci (Weinh). 2024 May 30;11(29):2308523. doi: 10.1002/advs.202308523 (PMC11304250; doi:10.1002/advs.202308523)
Supplement: Supplementary file 1 — Supporting Information [file ADVS-11-2308523-s001.pdf]

ADVANCED SCIENCE
Open Access

Supporting Information

for *Adv. Sci.*, DOI 10.1002/advs.202308523

Deuteration-Induced Energy Level Structure Reconstruction of Carbon Dots for Enhancing Photoluminescence

Zimin Yao, Xiaokun Wen, Xia Hong, Ran Tao, Feifei Yin, Shuo Cao, Jiayi Yan, Kexin Wang and Jiwei Wang**

Supporting Information of

Deuteration-induced energy level structure reconstruction of carbon dots for enhancing photoluminescence

Zimin Yao,[a] Xiaokun Wen,[b] Xia Hong,[b] Ran Tao,[a] Feifei Yin,[a] Shuo Cao,[a] Jiayi Yan,[b] Kexin Wang,[a,*] Jiwei Wang,[a,*]

a *College of Physics, Liaoning University, Shenyang, 110036, China*

b *Key Laboratory of UV-Emitting Materials and Technology (Northeast Normal University), Ministry of Education, Changchun, 130024, China*

E-mail: wangkexin@lnu.edu.cn (Kexin Wang); wangjiwei@lnu.edu.cn (Jiwei Wang).

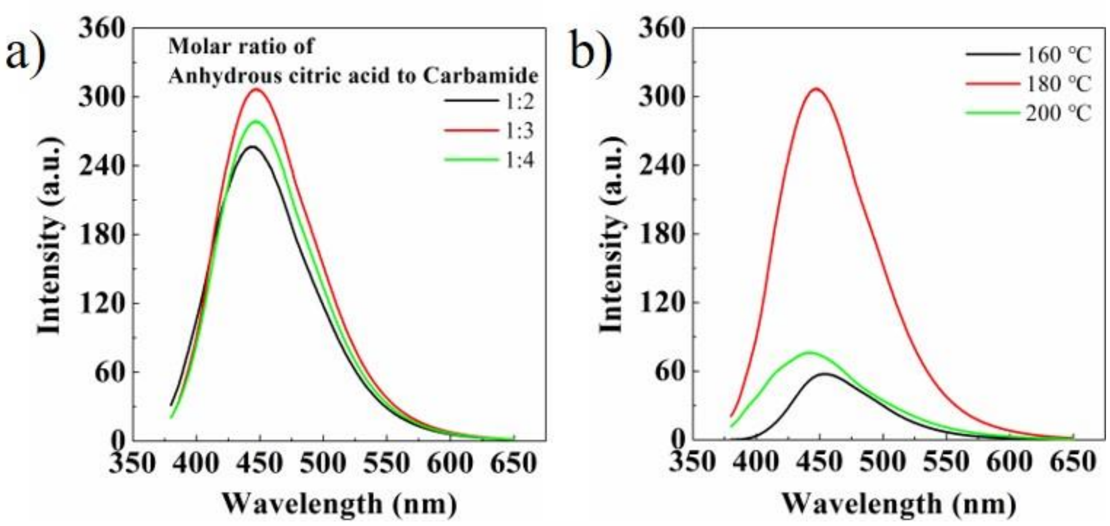

Figure S1. a) The PL spectra of CDs prepared from three different anhydrous citric acid to carbamide ratios; b) The PL spectra of CDs prepared from three temperatures.

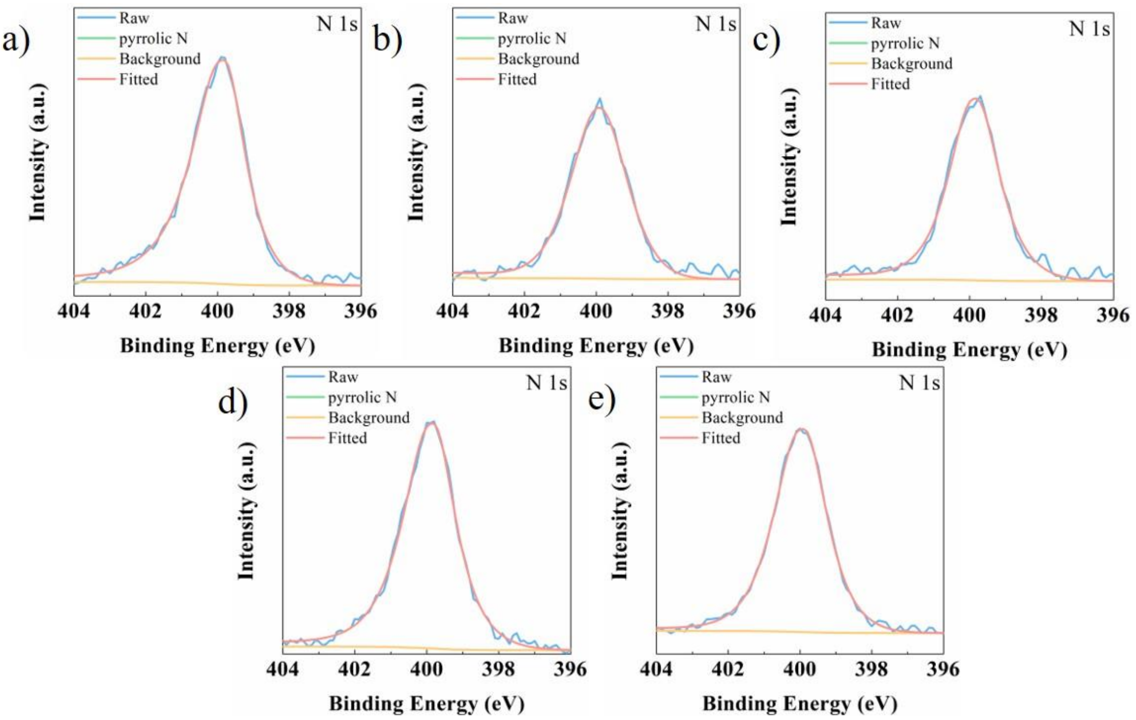

Figure S2. The high-resolution N1s XPS spectra of D_0-CDs a), $D_{0.2}$-CDs b), $D_{0.3}$-CDs c), $D_{0.5}$-CDs d) and D_1-CDs e), respectively.

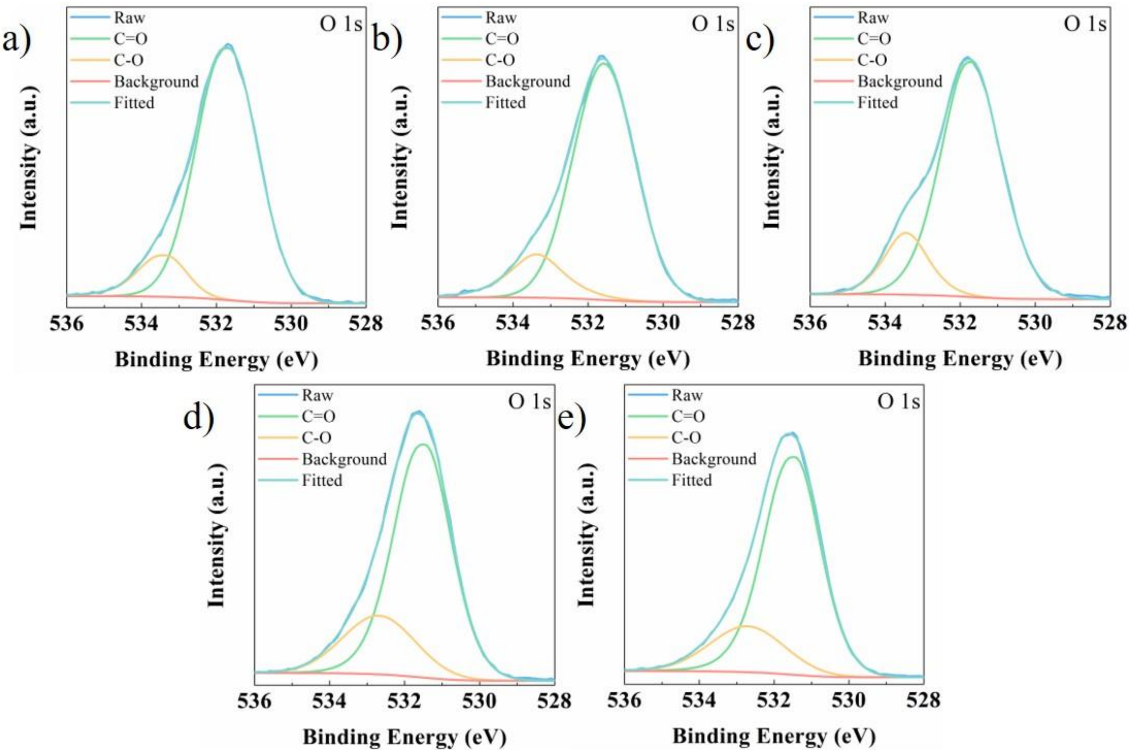

Figure S3. The high-resolution O1s XPS spectra of D_0-CDs a), $D_{0.2}$-CDs b), $D_{0.3}$-CDs c), $D_{0.5}$-CDs d) and D_1-CDs e), respectively.

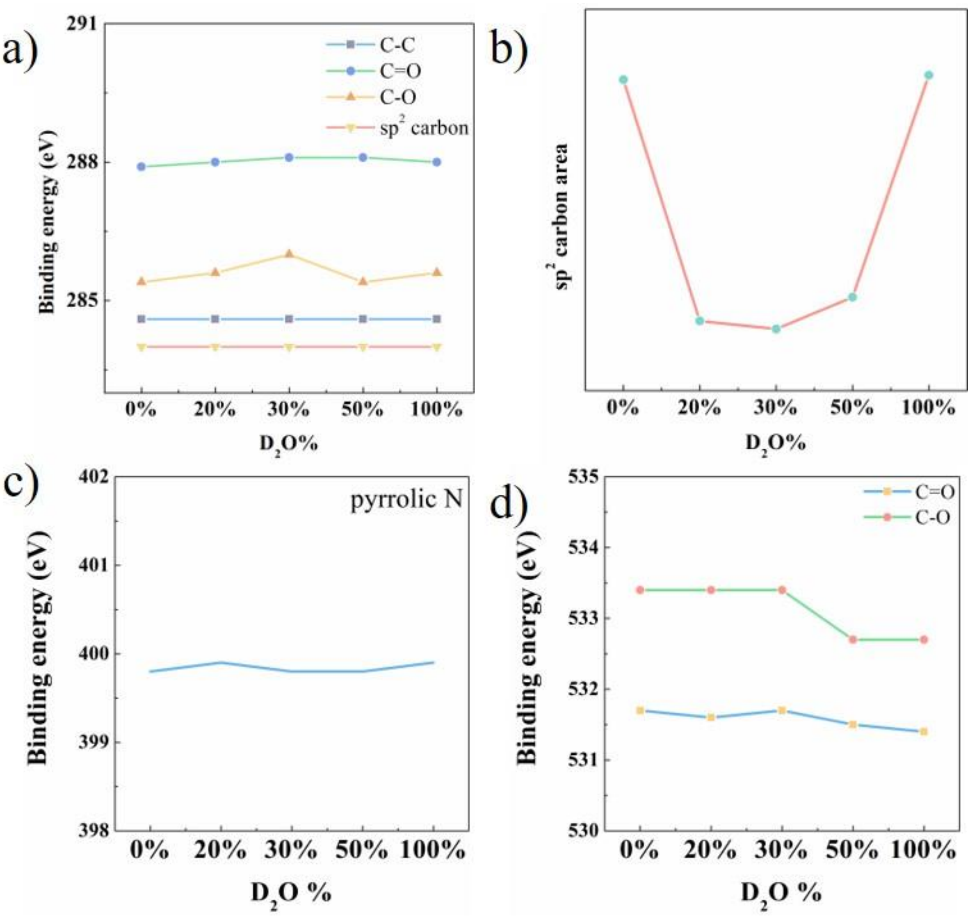

Figure S4. The change of binding energy about those C species (a); The band area variation of sp^2 carbon (b); The change of binding energy about those pyrrolic N (c); The change of binding energy about those O species (d).

Table S1. The quantum yields of CDs under the excitation at 280 nm and 390 nm.

	D_0-CDs	$D_{0.2}$-CDs	$D_{0.3}$-CDs	$D_{0.5}$-CDs	D_1-CDs
280 nm	9.9%	10.6%	12.2%	11.25%	10.6%
390 nm	10.9%	17.2%	25.3%	21.6%	16.1%
